# Dyspepsia and Consumption

**Published:** 1896-02-15

**Authors:** Solomon C. Smith

**Affiliations:** Physician to the Westminster General Dispensary.


### DYSPEPSIA AND CONSUMPTION.

By Solomon C. Smith, M.D., M.R.C.P., Physician to the Westminster General Dispensary.

### I.—DYSPEPSIA AS A CAUSE AND COMPLICATION.

The relation between digestive disorders and phthisis pulmonalis is too marked to escape observation. At every phase of its progress the question of the digestive capacity of the individual intrudes itself. However completely we accept the proofs which have been accumulated as to the bacillary origin of tuberculosis, we cannot but see that the thing called tubercle does not consist of tubercle bacilli, but is made up of altered tissue cells, more or less imperfectly organised by the individual for the individual's protection against the venomous intrusion.

It is impossible to shut one's eyes to the fact that in a very large number of cases tubercle bacilli establish themselves in the body, affecting glands or joints, or may be the lungs themselves in certain localised areas, and yet spread no further. The stuff called tubercle is, in fact, the outcome of a conflict between man and microbe, and in the encapsuled variety is but the dead battlefield, where the struggle has been fought and the victory won by which the larger organism has conquered and shut up the multitudes of its assailants within strict bounds.

Far more important, then, than the character of the intruding tubercle bacillus is the vigour and activity of the frame which it assails, for on this depends whether the cell growth by which the bacilli become surrounded shall encapsule them and render them harmless, or shall itself in turn become a fitting soil for their further development.

Now vigour, capacity for work, power of resisting evil influences, and all that we mean by the term vitality depends, in the ultimate analysis, upon the power possessed by the body of appropriating for its own purposes the energy lying latent in the food stuffs which it digests, and so we arrive at the great truth that not only life, but the power of resisting the attacks of other living organisms depends on the activity and perfection of the digestive processes.

It is a fact, then, worthy of the most careful consideration that not only is consumption often preceded by dyspepsia, so much so, that long before the existence of microbes was suspected, a pre-tubercular stage was described which was practically a stage of indigestion and innutrition, but that during the course of the disease its curiously erratic progress seems to depend even more upon varying digestive capacity than upon the climatic surroundings on which so much stress is often laid. In fact, one might go so far as to say that in cases of phthisis uncomplicated with bronchitis climate acts beneficially more by means of its effect upon appetite and digestion than by any direct influence it has upon the lung. We have then to consider dyspepsia in the light both of a cause and a complication of phthisis.

No doubt there was an apparent absurdity in speaking of a pre-tubercular stage of tubercle, and by many it was maintained, as it is now, that this stage was but one in which tuberculosis existed, but was not discovered. This, of course, is a position which it is impossible either to prove or to disprove; but it is worth noting that our increasing knowledge of the habits of the bacillus as the *vera causa* of tuberculosis makes the assumption of a pre-tubercular condition of the patient a necessity. Knowing the widespread nature of the infection, and the fact that the bacilli may actually exist within the body for long periods without giving rise to any general extension of the disease, it becomes necessary to assume the concurrence of some other condition, by the operation of which certain individuals and certain parts of those individuals, and certain periods of their lives, are selected by the disease. Such antecedent conditions we recognise in injuries of joints, in broncho-pneumonia and pulmonary collapse after whooping cough, and in the lowered vitality which occurs in those who are engaged in dark and crowded workshops. All these are accepted as so-called predisposing causes of tuberculosis, or as conditions which have rendered possible the intrusion of the bacillus.

Taken in this sense, the dyspepsia which so often precedes phthisis may truly be called a pre-tubercular stage, *i.e.*, a diseased condition which enables tuberculosis to develop, and without which it would not occur.

It is difficult, however, to accept the suggestion made by some that the dyspepsia, which in this sense causes phthisis, is in any way different from what may occur under ordinary circumstances; nevertheless, it is true enough that certain types of dyspepsia are much more apt than others to be followed by consumption, and that their occurrence in people who, either from heredity or surroundings, are predisposed to the disease, should therefore be looked upon with much suspicion, and should lead to more than mere drug treatment.

If it is possible, as we know it is, to arrest phthisis by changing the surroundings of the patient, not only after tubercle has been developed, but after it has caseated, and after cavities have formed, how much more possible must it not be to eliminate the tendency to tuberculosis when the bacillus has not yet even obtained a foothold in the body. Hence the importance of the early recognition and early treatment of all conditions which are known to be predisposing causes, and especially of that most frequent precursor of phthisis —dyspepsia.

In considering dyspepsia as a complication of phthisis, it must be remembered that too often it is the case that the condition of indigestion which has led up to the disease continues through its early stages, aggravating it in every way. Later on, however, new forms of dyspepsia are developed which are the direct products of the major malady. But whatever forms may be taken by the indigestions associated with phthisis, they are all important in the highest degree. They hold a chief place in determining the progress of the disease; they, far more than the diseased patches in the lungs, can be satisfactorily dealt with by well-devised treatment; and on their amenability to treatment, perhaps more than on any other point except the range of the pyrexia, depends the prognosis.

